# Innovative Blended Learning Curriculum in Noninvasive Ventilation for Pulmonary and Critical Care Fellows

**DOI:** 10.1055/s-0045-1811702

**Published:** 2025-09-26

**Authors:** Asil Daoud, Cassondra Cramer-Bour, Divya Venkat, Abdulghani Sankari

**Affiliations:** 1 Division of Pulmonary & Critical Care, Detroit Medical Center System, Wayne State University School of Medicine, Detroit, Michigan, United States; 2 Department of Medical Education, John D. Dingell VA Medical Center, Detroit, Michigan, United States; 3 Division of Pulmonary & Critical Care, John D. Dingell VA Medical Center, Detroit, Michigan, United States; 4 Department of Medical Education, Ascension Providence Hospital, Southfield, Michigan, United States

**Keywords:** noninvasive ventilation, hypoxic respiratory failure, hypercapnic respiratory failure, blended learning, graduate medical education

## Abstract

**Introduction:**

There is a lack of a standardized curriculum for the appropriate use of noninvasive ventilation (NIV), which is readily accessible. Management of NIV is a core competency for physicians training in pulmonary and critical care medicine (PCCM). We present a blended model of instruction that was highly successful in our pilot program.

**Methods:**

The curriculum targeted eight first-year PCCM fellows to assess knowledge and confidence in key competencies of NIV management. After a baseline assessment, fellows engaged in both hands-on instruction and traditional didactics in NIV. Following, fellows were encouraged to use the e-learning modules for enhanced instruction. The modules were designed to cover all major aspects of NIV management and with unique interactive patient cases for both inpatient and outpatient uses of NIV.

**Results:**

Eight first-year PCCM fellows completed the training and responded to the posttest assessment 4 weeks later. The average multiple-choice questions (MCQs) score increased from 13.5 ± 3.2 (54.0%) to 18.4 ± 1.6 (73.6%) and was significant ( *p* = 0.004). A Likert assessment of learner confidence also showed significant improvement across several key competency domains.

**Conclusion:**

This curriculum represents a successful and novel approach to NIV education, a critical but challenging core competency in pulmonary medicine for physicians training in PCCM.

## Introduction

Noninvasive ventilation (NIV) is a well-validated form of respiratory support for hypoxic and hypercapnic forms of respiratory failure in acute and chronic settings. 1 2 3 Use of NIV in patients with respiratory failure improves lung function, reduces hospital admissions, intubations, and is associated with improved mortality. 4 5 6 Use of NIV for respiratory failure is not always appropriate in all clinical settings. It is advisable to consider NIV as a tool that leads to the desired outcome if applied to the appropriate clinical and physiologic context, and if the appropriate equipment and appropriate monitoring are in place. Meeting each of those important steps in the best use of NIV is a core competency in training for pulmonary physicians and is tested by the American Board of Internal Medicine certification exam for pulmonary medicine.

Inadequate staff training has been associated with NIV failure. In a small prospective observational study of patients with acute respiratory failure (ARF) who were being treated with NIV, inadequate staff training was strongly associated with NIV failure ( *p* = 0.007). In this study, some of the errors detected included the following: poor mask fit with excessive air leaks, lack of training in recognizing ventilator alarms, and even simple ventilator setup. 7 Staff education may be one explanation for the notable divergence of the reported low mortality in ARF reported in literature versus that observed in the real-world clinical practice. 8 Though the use of NIV in acute and chronic respiratory failure is well established, many pulmonary and critical care medicine (PCCM) fellowship programs lack dedicated training curricula to achieve competency in this topic.

What should this curriculum look like? An international panel of experts has released a consensus statement with a “call to action” to improve educational resources regarding NIV for physicians in training. 9 This call to action also included recommendations for formulating such a curriculum, providing emphasis on the theory of NIV as well as key practical steps toward equipment setup, using a multimodality approach with internet-based resources and hands-on simulation, along with working closely with a senior expert in NIV.

Currently published curricula aimed at educating physicians in NIV have focused on simulation. 10 11 Simulation is a highly effective tool in medical education, but it is also associated with a high startup cost and does not have the flexibility of online resources that trainees can turn to repeatedly. Other efforts to address this problem have included proprietary online resources that are not widely accessible to all. 12 For this reason, we were interested in structuring a curriculum that would represent a blended model of both online and in-person hands-on instruction wherein the online component is freely available for all. Online learning or “e-learning” can effectively teach hands-on skills and has even been used successfully to improve novice surgeons' laparoscopic cholecystectomy technique. 13 14 15 Blended learning using a combination approach, where a component of instruction occurs synchronously in person and is coupled to asynchronous online resources, is used by the learners in a complementary fashion. Blended learning encourages self-directed learning, enhances learner self-efficacy, and is effective for graduate medical learners. 16 17

Our objective was to devise a curriculum that leverages impactful online resources to augment face-to-face instruction for physicians training in pulmonary and critical care. This initiative aims to equip these physicians with the necessary skills to provide superior quality care to patients requiring noninvasive ventilatory support.

## Methods

### Learner Selection

The target learner group included first-year fellows in critical care and pulmonary and critical care ( *N* = 8) at a single large tertiary care hospital. The curriculum was implemented during fellow orientation, prior to starting any clinical rotation. This study was declared exempt by the Wayne State University (WSU) Institutional Review Board Administration Office.

### Curriculum Development

The learning objectives for this curriculum were designed by a panel of experts in graduate medical education, PCCM, and sleep medicine. The e-learning modules were developed using an online platform, which generated high-quality animated characters representing intensivists and pulmonologists in inpatient and outpatient settings, along with nurses, respiratory therapists, and patients. The goal of these animated videos was to create life-like clinical scenarios focusing on the use of NIV in ARF in the intensive care unit (ICU) and chronic respiratory failure in the outpatient setting. Then, these animated videos were uploaded to the website Edpuzzle.com, which is a free-to-use video platform with wide availability. Embedded in the videos are interactive questions aimed at answering the learning objectives and promoting an active learning style.

### E-Learning Module Design

The curriculum consisted of four e-learning modules ( Appendix A ). The first module (17:30 minutes in length) introduced NIV. It contained a detailed explanation of the breath cycle, ventilation, oxygenation, and pathophysiology behind using NIV in respiratory failure and sleep. It also included general recommendations on initial NIV settings, NIV titration, optimal patient selection, contraindications to use of NIV, how to wean from NIV, and, finally, signs of NIV failure. This module also included a detailed overview of a typical NIV machine interface, including patient setup, how to assess mask fit, and knobology. In the second module (11:07 minutes in length), we presented a case of a patient with acute hypercapnic respiratory failure from underlying chronic obstructive lung disease (COPD). The learner assesses forms of respiratory support, titrates the NIV based on patient laboratory data, optimizes the patient/mask interface, and finally assesses for NIV failure. In the third module (11:08 minutes in length), we present a case of chronic hypercapnic respiratory failure from COPD. Here, the learner assesses indications for NIV in the outpatient setting, key phrases for how to elicit history from the patient to assess for dyssynchrony, and how to monitor compliance with downloaded device reports. The fourth module (run time of 5:57 minutes) includes a detailed explanation of the diverse types of dyssynchrony during a breath cycle and a recommended approach for how to improve it. These dyssynchronies were illustrated with ventilator waveforms and the expected clinical signs and symptoms of the patient. These modules are further described in Table 1 .

**Table 1:** Module outline with learning objectives

Content	Learning objectives
Module 1: Introduction to NIV	1. Understand the definition of NIV 2. Identify NIV equipment, interface, and knobology 3. Review physiology of ventilation and terminology in NIV 4. Summarize common indications of NIV, and appropriate inclusion and exclusion criteria
Module 2: NIV use in the inpatient setting: acute hypercapnic respiratory failure	1. Diagnose acute COPD exacerbation based on presenting signs and symptoms 2. Identify NIV as appropriate respiratory support 3. Suggest initial NIV settings based on clinical context 4. Formulate a plan for patient management using a clinical scenario 5. Troubleshoot common NIV alarms 6. Assess for failure of NIV and appropriate weaning strategies
**Module 3:** NIV use in the outpatient setting: chronic hypercapnic respiratory failure	1. Evaluate the role of NIV in chronic hypercapnic respiratory failure 2. Suggest initial NIV settings based on clinical context 3. Appraise the role of sleep study and end-tidal CO _2_ in titration of NIV settings 4. Recognize sleep study hypnogram and adherence report interpretation 5. Troubleshooting of common NIV patient complaints to improve adherence
Module 4: NIV dyssynchrony	1. Recognize diverse types of NIV dyssynchrony based on waveform analysis 2. Plan appropriate interventions to flow, tidal volume, trigger, and mode to treat dyssynchrony

Abbreviations: COPD, chronic obstructive pulmonary disease; NIV, noninvasive ventilation.

### Assessment

Learners were first given a pre-test questionnaire to assess baseline knowledge prior to participating in the curriculum. This test consisted of 25 multiple-choice questions (MCQs) to assess cognitive skills and medical knowledge on NIV use in the different settings of ICU, general wards, and outpatient pulmonary clinics (see Appendix A , available in the online version only). These MCQs were created using the most recent evidence-based guidelines and practices published on NIV and approved by our panel of experts involved in curriculum design. The backgrounds of this panel included physicians board-certified in sleep medicine and pulmonary medicine with between 2 and 16 years of clinical practice experience. Additionally, a pretraining confidence survey was created using a 5-point Likert scale used for subjective evaluation of comfort level using NIV in different practice settings.

### Blended Model of Instruction

Following completion of the questionnaire, the learners then participated in a 1-hour didactic with a senior chief PCCM fellow and board-certified sleep medicine physician. This didactic focused on unique indications for NIV not addressed in the online modules detailed earlier. Following this, the learners then engaged in 30 minutes of hands-on instruction in the practical aspects of how to set up NIV for a patient with a certified respiratory therapist. The learners were then given access to the e-learning modules detailed above. The fellows were strongly encouraged to utilize this resource, but it was not required. To assess for longer-term knowledge retention, a 4-week posttest was conducted.

## Results

**Fig. 1:**
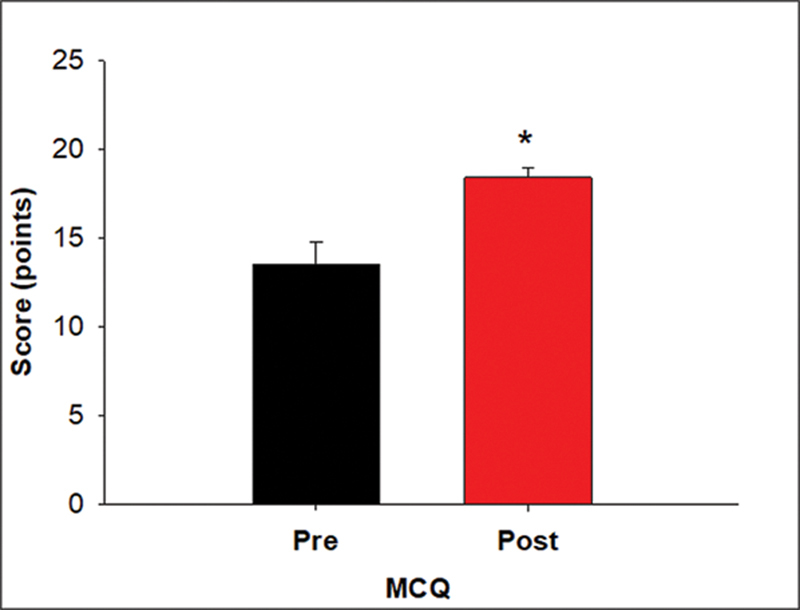
Graph depicting improvement in the mean scores for the MCQ-based knowledge test after 4 weeks of implementation of the noninvasive ventilation e-curriculum ( *p* = 0.004). MCQ, multiple-choice questions.

In our pilot study of a blended model of NIV curriculum, we describe results for eight first-year fellows, six PCCM and two critical care medicine. We had 100% participation in the posttest assessment conducted at 4 weeks ( *N* = 8). The assessment included MCQs for a knowledge assessment and Likert scales assessing confidence in key management steps of NIV. The average MCQ score increased from 13.5 ± 3.2 (54.0%) to 18.4 ± 1.6 (73.6%), which was significant ( *p* = 0.004; Fig. 1 ). The Likert assessment of learner confidence in managing NIV also revealed improved scores for all competencies ( Tables 1 and 2 ).

**Table 2:** Mean and standard deviation of baseline and post-training confidence survey results of core competencies tested on curriculum assessment, using a 5-point Likert scale with 5 as extremely comfortable, 1 as not comfortable at all

NIV competency subject	Pretraining mean ± SD	Posttraining mean ± SD	*p* -Value
NIV management in acute COPD exacerbation	3.1 ± 0.8	4.4 ± 0.5	*0.002*
NIV management in pulmonary edema	3.3 ± 0.7	4.1 ± 0.6	*0.041*
Appropriateness for NIV initiation in the ICU	2.5 ± 0.9	4.6 ± 0.5	*0.016*
Identification of the best NIV mode by clinical scenario in the ICU	2.5 ± 0.9	4.1 ± 0.6	*< 0.001*
Titration of NIV settings in the ICU by clinical scenario	2.3 ± 0.9	4.5 ± 0.5	*< 0.001*
Liberation from NIV in the ICU by clinical scenario	3.1 ± 1.1	3.6 ± 1.2	0.487
Management of NIV in chronic hypercapnic respiratory failure	2.8 ± 1.0	4.1 ± 0.6	*0.008*
Appropriateness for initiation of long-term NIV in an outpatient setting	2.6 ± 1.1	4.0 ± 0.5	*0.008*
Identification of the best NIV mode by clinical scenario in an outpatient setting	2.5 ± 1.1	4.0 ± 0.5	0.063
Titration and management of NIV in an outpatient setting	2.3 ± 0.9	4.3 ± 0.4	*< 0.001*
Troubleshooting of NIV alarms and mask fit issues	2.3 ± 1.0	4.3 ± 0.9	*0.003*

Abbreviations: ICU, intensive care unit; NIV, noninvasive ventilation.

Note: Values in italics indicate statistical significance.

## Discussion

We present a successful curriculum of NIV, which covers all major aspects of appropriate use of NIV in common clinical scenarios and addresses a core competency in training pulmonary and critical care physicians. Unlike other published curricula, our educational tool does not require any specialized equipment for setup or proprietary online resources. We used a blended model of instruction where our learners engaged in traditional didactic models of instruction in core concepts of NIV and utilized highly detailed e-learning modules for self-directed learning. The e-learning modules were vetted by our panel of experts and can stand alone as their own educational tool should other programs be interested in expanding their own curriculum in NIV management and do not have dedicated faculty support for hands-on instruction. The e-learning modules are hosted on Edpuzzle.com, which is a free-to-use platform available wherever there is an internet connection.

Our findings show a significant increase in longer-term knowledge assessed by improved knowledge scores at a 4-week time point (54.0–73.6%), and significant improvement in several NIV competencies. Moreover, this is the first curriculum that uses a blended modality of instruction with e-learning and hands-on instruction, which can be scalable to other programs with limited resources. However, our curriculum does have limitations that are important to consider. This curriculum was piloted for our incoming classes of fellows ( *N* = 8), and it is noteworthy to consider how the findings may or may not be replicated in larger groups. Our learner group included incoming fellows during orientation, where they are exposed to lots of incoming information, though we assumed no prior knowledge of NIV. Inherent in our assessment strategy is the use of pre- and posttest questionnaires, which are subject to biases such as the maturation effect and testing effect, which may augment the positive outcome of the curriculum seen in our study. It is unknown to what extent the learners engaged in additional study on the topic of NIV or relevant clinical exposure following the hands-on lecture and the eventual posttest 4 weeks later, which may have an independent effect on the significantly improved scores. Our assessment strategy is optimized to evaluate improved knowledge, key NIV competencies, and confidence in NIV, but it is not optimized to assess bedside skills application, which is another limitation. This could be addressed through a standardized, observed, structured clinical examination or through case logs of NIV bedside titration with attending cosignature.

In summary, our curriculum represents a successful approach to blended learning instruction on comprehensive NIV management for the pulmonary and critical care fellow in training.
