# Supplementary material for: Innovative Blended Learning Curriculum in Noninvasive Ventilation for Pulmonary and Critical Care Fellows
Source: Avicenna J Med. 2025 Sep 26;15(3):131–5. doi: 10.1055/s-0045-1811702 (PMC12520753; doi:10.1055/s-0045-1811702)
Supplement: Supplementary file 1 — Supplementary Material [file 10-1055-s-0045-1811702-s240162.pdf]

Appendix A

Edpuzzle online modules with links

Class Name: NIV made easy
Classroom Code: jifefac
Module 1: Introduction to NIV
https://edpuzzle.com/media/626d8ad7224ae34298dc5b02
Module 2: Use of NIV in acute COPD in ICU setting
https://edpuzzle.com/media/626d8c93a043bc42ba27cb7f
Module 3: Use of NIV in chronic COPD
https://edpuzzle.com/media/626d9abdfcb03f42cf5ad5a3
Supplemental Module: Dyssynchrony https://edpuzzle.com/media/628a4a36a8534040fbfe6bcc

 © 2025. The Author(s).

Multiple-choice questions (MCQs) on noninvasive ventilation (NIV)

Q1: Label the four phases of the respiratory cycle shown in ►Fig. 1:

1) 1: trigger, 2: inspiration, 3: cycle, 4: expiration.
2) 1: inspiration, 2: trigger, 3: cycle, 4: expiration.
3) 1: cycle, 2: inspiration, 3: trigger, 4: expiration.

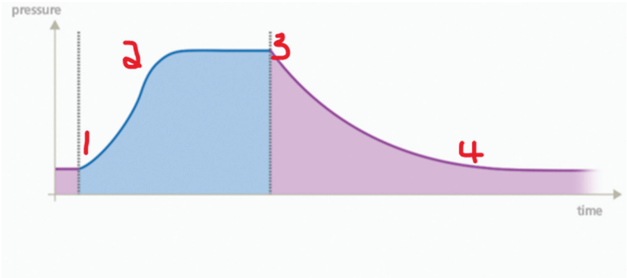

Fig. 1

Q2: What does the term pressure support (PS) indicate when used in noninvasive ventilation?

1) Pressure support (PS) = Inspiratory positive airway pressure (IPAP) – Expiratory positive airway pressure (EPAP).
2) Pressure support (PS) = Inspiratory positive airway pressure (IPAP) + Expiratory positive airway pressure (EPAP).
3) Pressure support (PS) = Inspiratory positive airway pressure (IPAP)/Expiratory positive airway pressure (EPAP).

Q3: The best way to describe how continuous positive airway pressure (CPAP) works in COPD:

1) CPAP adds extrinsic PEEP to intrinsic PEEP to generate the breath.
2) CPAP counterbalances the inspiratory pressure load of intrinsic PEEP, thus decreasing the work of breathing.
3) CPAP supports ventilation by providing positive pressure.

Q4: What modes of noninvasive ventilation (NIV) require spontaneously breathing patient:

1) CPAP.
2) BPAP S mode.
3) BPAP ST mode.
4) Pressure assist control (PAC).
5) 1 + 2.
6) All forms of NIV require spontaneously breathing patients.

Q5: Clinical vignette:

A 65-year-old male with a medical history of COPD on 2LPM O2 at home comes to the emergency department complaining of dyspnea and wheezing for a few days.

Vitals: blood pressure 140/80, HR 90, RR 22, SpO_2 88%, weight 80 kg.

Exam showed the patient tachypneic with expiratory wheezing.

ABG: PH 7.16, CO_2 75, PaO_2 55.

What is the interpretation of the above-mentioned blood gas?

1) Acute hypoxic hypercapnic respiratory failure.
2) Acute on chronic hypoxic hypercapnic respiratory failure.
3) Chronic hypoxic hypercapnic respiratory failure.

Q6: For the patient mentioned in the clinical vignette in Question 5, you decide to start non-invasive ventilation for acute hypercapnic respiratory failure and work of breathing. What would you suggest as the initial settings if you know the patient is about 80 kg?

1) Start with CPAP of 10 cm H_2O, FiO_2 100% to correct hypoxemia, and then titrate afterward based on clinical response and by obtaining frequent blood gases.
2) Start with IPAP of 10 cm H_2O and EPAP of 5 cm H_2O with FiO_2 of 100%, and titrate afterward based on clinical response and by obtaining frequent blood gases.
3) Start with IPAP of 16 cm H_2O and EPAP of 8 cm H_2O with a backup rate of 12 bpm, titrate afterward based on clinical response and by obtaining frequent blood gases.

Q7: Clinical vignette:

A 70-year-old male in the ICU was intubated for acute hypoxic hypercapnic respiratory failure.

His current vent settings are: 14/400/40/5.

ABG: 7.38/55/80/25.

Vitals: BP: 130/80, HR: 80, RR 18, Sat 98%.

His rapid shallow breathing index (RSBI) is 45.

He is following commands and off sedation and asking to be extubated.

What would be the best next step?

1) Extubate to room air (RA)/high-flow nasal cannula (HFNC).
2) Extubate to NIV.
3) Keep intubated for now, SBT again the next day.

Q8: Clinical vignette:

A 70-year-old patient who was originally intubated for acute CHF exacerbation was extubated 6 hours ago. He was

 © 2025. The Author(s).

doing okay for the first 2 hours, but then noted to be more tachypneic, using accessory muscles, and diaphoretic.

His vitals are: BP 180/120, HR 120, RR 30, Sat 91% on 10 L O_2.

What would you do?

1) Trial of NIV.
2) Trial of NRB mask.
3) Intubate now.
4) Trial of HFNC.

Q9: Clinical vignette:

A 62-year-old female presented to the ED complaining of cough, sputum production, and SOB.

Vitals: BP 120/70, HR 92, RR 24, Sat 88% on RA.

Exam showed bilateral rhonchi and diminished breath sounds on the right > left.

CXR showed bilateral infiltrate, more prominent on the right side, concerning multifocal pneumonia.

She received one bolus of IV fluids and was started on antibiotics in the emergency department. The patient was then placed on NIV for work of breathing, and ICU was consulted for further recommendations.

What would you suggest doing?

1) Intubate the patient.
2) Continue trial of NIV and assess response, including oxygen saturations and work of breathing.
3) Supplemental O_2/HFNC if needed to keep $SpaO_2$ >92%.

Q10: Clinical vignette:

A 55-year-old female with a history of myasthenia gravis came to ED complaining of fatigue and tiredness, dyspnea, and diplopia. Vitals showed BP 120/70, HR 98, RR 26, SpO_2 88% on RA.

CXR showed no infiltrates.

Sepsis workup was sent.

Bedside FVC was 1.5 L and MIP was –60

What would you do next?

1) Supplemental O_2 to keep $SpaO_2$ >92%.
2) Intubate the patient for impending respiratory failure.
3) Trial of NIV and frequently assess the patient.

Q11: What value on noninvasive ventilation helps you with oxygenation?

1) EPAP and FiO_2.
2) IPAP.
3) IPAP-EPAP.
4) FiO_2

Q12: What value on noninvasive ventilation (NIV) helps you with ventilation?

1) EPAP and FiO_2.
2) IPAP.
3) Pressure support, which is IPAP-EPAP.

Q13: Please identify the type of desynchrony illustrated in ►Fig. 2.

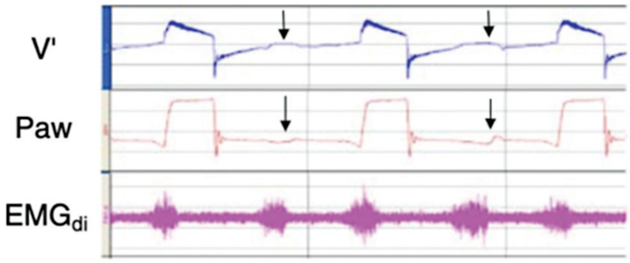

Fig. 2

1) Ineffective triggering.
2) Double triggering.
3) Premature cycling.
4) Late cycling.

Q14: Please identify the type of desynchrony illustrated in ►Fig. 3.

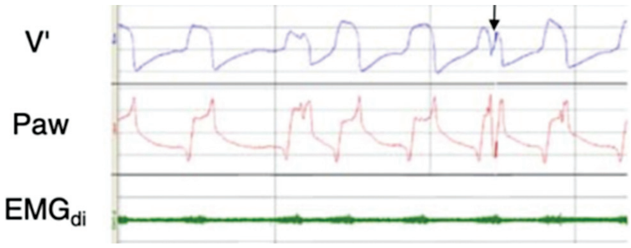

Fig. 3

1) Late cycling.
2) Ineffective triggering.
3) Premature cycling.
4) Double triggering.

Q15: Please identify the type of desynchrony illustrated in ►Fig. 4.

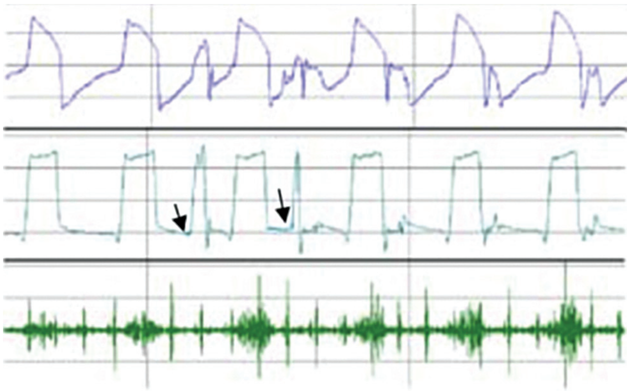

Fig. 4

1) Premature cycling.
2) Ineffective triggering.
3) Double triggering.
4) Late cycling.
5) Auto-triggering.

Q16: Please identify the type of desynchrony illustrated in ►**Fig. 5**.

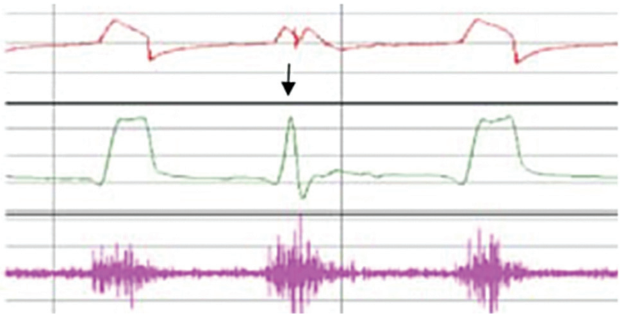

Fig. 5

1) Ineffective triggering.
2) Premature cycling.
3) Double triggering.
4) Late cycling.

Q17: Please identify the type of desynchrony illustrated in ►**Fig. 6**.

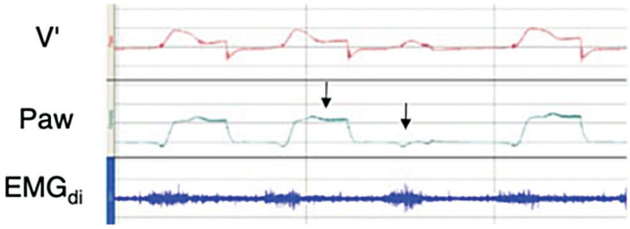

Fig. 6

1) Double triggering and premature cycling.
2) Ineffective triggering.
3) Premature cycling and auto-triggering.
4) Late cycling and ineffective triggering.

Q18: The time in which the inspiratory pressure reaches the target pressure of IPAP from the onset of the respiratory phase:

1) Ti min
2) Flex time
3) Ti max
4) Rise time

Q19: Choose all that apply:

What parameters on the noninvasive ventilation ensure adequate time is spent in inspiration?

1) Cycle sensitivity.
2) Rise time.
3) Ti min.
4) Ti max.
5) Pressure support.
6) IPAP.

Q20: Which of the following is an *absolute* contraindication to start noninvasive ventilation (NIV)?

1) Esophageal perforation.
2) GCS <8 secondary to hypercapnia.
3) Excessive respiratory secretions.
4) Presence of acidosis with PH 7.25.

Q21: How can you reduce (troubleshoot) late cycling on NIV?

1) Decrease rise time.
2) Decrease Ti max.
3) Decrease Ti min.
4) Decrease respiratory rate.
5) Decrease pressure support.

Q22: For patients with recent hospitalization for acute hypercapnic respiratory failure related to acute exacerbation of COPD (AECOPD) requiring noninvasive ventilation (NIV), the ATS recommends:

1) Follow up in clinic in 1 to 2 weeks after discharge to evaluate the need for NIV.
2) Starting NIV upon discharge from the hospital and follow-up in the clinic in 1 to 2 weeks.
3) Follow up with a sleep study for PAP titration to obtain the best results.

Q23: What is false about the criteria of obesity hypoventilation syndrome (OHS) diagnosis?

1) Patients with a BMI $\geq 30\,kg/m^2$.
2) Presence of daytime alveolar hypoventilation (awake, sea-level, arterial $PcO_2 > 45$ mm Hg).
3) The absence of other causes is not needed for diagnosis if the first two points are achieved.
4) $1+2$.

Q24: What is the trigger on NIV?

1) The ventilator detects a change in flow, which triggers the ventilator to change from expiration to inspiration pressure.
2) The ventilator detects a change in pressure, which triggers the ventilator to change from expiration to inspiration pressure.
3) The ventilator detects a change in volume, which triggers the ventilator to change from expiration to inspiration pressure.
4) The ventilator detects a change in time, which triggers the ventilator to change from expiration to inspiration pressure.
5) All the above.

 © 2025. The Author(s).

Q25: Clinical vignette:

A patient with a medical history of chronic hypercapnic respiratory failure and COPD who was started on noninvasive ventilation, with empirical settings of 18/8/30% after recent hospitalization with AECOPD, presents to the pulmonary clinic for follow-up. Patient reports being comfortable on the mask with no issues sleeping with it; what would be the next step?

1) Continue the same settings as long as the patient is comfortable with the mask.
2) Refer the patient does a PAP titration to target the PCO_2 of a normal value.
3) Refer the patient for PAP titration to target reduction of 5 to 10 mm Hg of PCO_2 seen on recent admission.
4) Obtain blood gas and titrate BPAP in the clinic based on PCO_2.

Q26: Clinical vignette:

A patient with a medical history of chronic hypercapnic respiratory failure and COPD who was started on noninvasive ventilation, with empirical settings of 18/8/30% after recent hospitalization with AECOPD, presents to the pulmonary clinic for follow-up.

Patient reports nonadherence to BPAP due to waking up with nasal congestion and dry mouth in the morning. Patient uses a nasal mask.

What would you suggest doing?

1) Try a nasal spray.
2) Try a full face mask.
3) Increase humidification.
4) Try adding a chin strap to the nasal mask.
5) All of the above.

 © 2025. The Author(s).
